# Factors associated with HIV infection and the utilization of HIV testing services among transgender people in Georgia

**DOI:** 10.1371/journal.pgph.0004956

**Published:** 2025-07-22

**Authors:** Maka Gogia, Mark H. Kuniholm, Pavlo Smyrnov, Jack DeHovitz, Tamar Zurashvili, Mamuka Djibuti

**Affiliations:** 1 Ivane Javakhishvili Tbilisi State University, Tbilisi, Georgia; 2 Partnership for Research and Action for Health, Tbilisi, Georgia; 3 Georgian Harm Reduction Network, Tbilisi, Georgia; 4 Department of Epidemiology and Biostatistics, University at Albany, Rensselaer, New York, United States of America; 5 Alliance for Public Health, Kiev, Ukraine; 6 State University of New York, Downstate Health Sciences University, Department of Medicine, Brooklyn, New York, United States of America; New York University, UNITED STATES OF AMERICA

## Abstract

We assessed factors associated with HIV infection as well as the utilization of HIV testing among transgender people (TGs) in the country of Georgia. From July 2020 to January 2021, TG participants were recruited using snowball sampling in three major Georgian cities: Tbilisi, Batumi and Kutaisi. The participants underwent structured face‒to-face interviews and rapid tests for HIV. The study employed descriptive analyses, and bivariate and multivariable logistic regression to explore factors associated with HIV infection and HIV testing history. Of the 95 participants, 49.5% identified as transgender women, 7.3% as transgender men, and 43.2% as non-binary individuals, with a mean age of 27.24 years (SD = 8.52). Overall, HIV prevalence was 24.1%, with transgender women experiencing the highest burden (40.5%). HIV prevalence was higher among transgender women (40.5%) and those involved into commercial sex work (41.7%), however, none of the predictor variables reached the level of significance in adjusted model. HIV testing in the past six months were reported by 76.8% of participants and predictors for testing were living alone (aOR=5.9, 95% CI: 1.06–32.69) and experiences of enacted stigma (aOR=1.76, 95% CI: 1.04–2.97). Conclusion: Our study reveals a high HIV burden among transgender individuals in Georgia, particularly transgender women. This is combined with significant gaps in the utilization of HIV prevention services. Further research is needed to explore the intersection of stigma and other barriers affecting a low uptake of HIV testing to inform the development of effective targeted interventions.

## Introduction

Transgender (TG) people are individuals whose gender identity does not align with the sex assigned to them at birth. Gender identity is distinct from both biological sex and sexual orientation. Globally, TG populations are disproportionately impacted by the HIV pandemic due to a complex interplay of individual, interpersonal, social, and structural factors. At the individual level, susceptibility to HIV is increased by behavioral risk factors such substance use, engagement in sex work, and condomless sex [1,2]. Access to health care is further restricted by interpersonal issues, such as experiences of abuse, stigma, and discrimination in social, familial, or medical contexts [3]. Social determinants, such as poverty, homelessness, and lack of education, exacerbate these vulnerabilities [4], while structural barriers, including restrictive legal policies and lack of trans-sensitive healthcare services, hinder effective HIV prevention, diagnosis, and treatment efforts [5]. However, the prevalence of HIV infection among TG varies widely by geographic region. For example, a recent meta-analysis estimated the HIV prevalence in transgender women to be 29.9% in Africa, 17.1% in the United States and Western Europe, and 13.5% in low and middle-income (LMIC) Asian countries [6]. In contrast, global HIV prevalence among transgender men is estimated at 2.6%, with regional variations. Data on HIV prevalence among non-binary individuals are currently limited [7], highlighting the need for further research in this area.

The prevalence of HIV among TG individuals has never been estimated in the country of Georgia, where the HIV epidemic is concentrated among men who have sex with men (MSM) (HIV prevalence 21.5%) [8], people who inject drugs (PWID) (HIV prevalence 0.9%) [9] and female sex workers (FSWs) (HIV prevalence 1.3%) [10]. Since 2012, heterosexual transmission has become the major route of transmission. This includes sexual transmission from current and former PWID to their non-injecting partners, as well as from MSM to their female sex partners. Analysis of national data on fast-track 90–90–90 targets in Georgia revealed that the significant gap in the cascade of the HIV care continuum was at the stage of HIV diagnosis, with 60% of the estimated number of people living with HIV aware of their status in 2018 [11]. This gap is larger for some key population (KP) groups, e.g., according to the same study, the coverage with HIV testing among MSM was 52.1%; however, such a gap has never been quantified among TG in Georgia.

The aim of this study was to assess HIV prevalence and identify factors associated with the uptake of HIV testing services among transgender people in Georgia. By addressing individual, interpersonal, social, and structural factors influencing HIV testing, the study seeks to inform the development of locally relevant targeted interventions to improve the first step of HIV care cascade among TG in Georgia as well as in the EECA region.

## Methods

### Participants and recruitment

We conducted secondary analyses of the study “Reaching new clients of trans community through peer driven intervention in Tbilisi, Georgia” conducted by Georgian Harm Reduction Network in 2020 [12]. The original study focused on outreach and service delivery with basic descriptive data, while our analysis explores factors associated with HIV infection and the utilization of HIV testing services among transgender individuals—questions not examined in the initial study.

Participants were recruited from three cities in Georgia—Tbilisi, Batumi, and Kutaisi—between July 2020 and January 2021 using exponential non-discriminative snowball sampling. Recruitment began with a convenience sample of three initial participants (seeds) who referred others from their social networks. Each seed provided multiple referrals, and the recruited participants, in turn, referred additional individuals. All referred participants were included in the sample. Participants received a 25 GEL (equivalent of 9€) incentive for completing the interview, but no additional incentives were provided for recruiting others. Nonmonetary incentives included free HIV, HBV, HCV, and syphilis testing and counseling. Recruitment continued until no additional participants could be identified within the study timeframe.

### Inclusion criteria

Eligibility criteria were self-identification as transgender, being 18 years of age or older, living in Tbilisi, Batumi and Kutaisi), proficiency in both speaking and reading Georgian and having a valid peer recruitment coupon. While the original study required participants to be living in Tbilisi, Batumi, or Kutaisi at the time of recruitment, no minimum duration of residence was set. However, peer referrals and recruitment were conducted through local networks, which naturally favored individuals with an established presence in these cities.

### Ethical considerations

The original study was approved by the Institutional Review Board (IRB) of the Health Research Union (a local research institution with an NIH/USA-certified IRB, NIH Registration: IORG0005619) in Tbilisi, Georgia (date of approval 30.04.20 valid until 30.04.21). All participants provided written informed consent before data. No research procedures took place until approval from the ethical review boards had been granted. Additionally, the Institutional Review Board of the Georgian National Centre for Disease Control and Public Health (certificate IRB00002150) approved the secondary analyses of the study results. Data access for the secondary analysis began on 18/03/2024.

### Data collection

After recruitment, two social/outreach workers conducted face-to-face interviews with a structured questionnaire. The interviewers were trained on ethical and sensitive issues related to the study. To facilitate the privacy and safety of the study participants, data collection was conducted in community-based settings that provide HIV prevention services to the lesbian, gay, bisexual, transgender, and queer (LGBTQ) population in a private environment without the presence of a third party. Informed consent was obtained from all the study participants.

Data collection took approximately 45 min. The structured questionnaire included sections about respondents’ general background and risky sexual practices, including engagement in commercial sex, drug use, discrimination, violence, and types of medical services received.

After completing the structured interviews, all participants were offered to voluntarily undergo rapid testing for HIV and HCV, HBV and syphilis by using World Health Organization (WHO) prequalified SD BIOLINE rapid test systems. In addition, national protocols on voluntary counseling and testing and screening protocols for rapid testing and infection control measures were followed. Pretest counseling, as an essential component of the HIV testing process, was offered before screening to ensure that the participants understood the implications of the test, potential results, and available resources. Both negative and positive test results were reported directly to study participants during posttest counseling, with the aim of providing support and guidance to them. In the case of HIV-positive results, participants were referred for confirmatory testing with referral forms to the National AIDS center. Those with positive HCV and syphilis tests were referred for free diagnostics, treatment and care services within the state program. In the case of negative results, posttest counseling focused on infection prevention and control messages. When the testing result was indeterminate, retesting was recommended, and such results were stored in the data register as “testing results that need retesting”.

### Definition of variables

*HIV infection* status was determined based on the results of HIV testing conducted through this study and classified as HIV positive or HIV negative;

*HIV testing history* - Participants were asked about their HIV testing experience within the last 6 months to assess recent testing behavior.

*Sociodemographic characteristics:*

Age – recorded as a continuous variable.
Gender identity – categorized as transgender women, transgender men, and non-binary individuals. Transgender women were defined as individuals who were assigned males at birth but identify as females. Transgender men were defined as individuals who were assigned female at birth but identify themselves as male. Non-binary individuals were defined as those whose gender identity does not align strictly with the traditional categories of male or female [13]. This included individuals assigned female or male at birth who identify outside of these binary categories.
Education level – classified into two groups: lower education level, including primary, secondary, or vocational education; and higher education level, indicating college or university education;
Employment status – categorized as: employed, which included participants engaged in full-time or part-time work, self-employment, business ownership, or casual/informal work; and unemployed, which included participants who were unemployed, retired, or on a pension;
Place of residence – assessed based on having a permanent place of residence over the past 3 months (dichotomized as “yes” or “no”);
Living arrangements – measured by whether participants were living alone (dichotomized as “yes” or “no”);
Income level – categorized into two groups (<500 GEL and ≥500 GEL).
Experiences of stigma, discrimination, and violence based on gender identity - assessed using the following 12 items: (1) experience of psychological pressure; (2) experience of insult and humiliation, including swearing, criticism, and derogatory nicknames; (3) experience of blackmail, including extortion, outing, threats, and intimidation; (4) experience of injury or physical pain, including bruising, strangulation, kicking, and other forms of physical harm; (5) experience of physical pursuit; (6) failure to receive necessary medical care; (7) denial of employment; (8) coercion to engage in sex without a condom; (9) experience of sexual violence or an attempted rape; (10) experience of rape; (11) coercion to engage in sexual acts considered unacceptable (including those involving physical violence, group sex, or other non-consensual forms); and (12) forced use of drugs or alcohol. Each of these experiences was assessed within the past 12 months, with responses dichotomized as either “yes” or “no. To quantify enacted stigma, we created an enacted stigma score by summing the number of “yes” responses across all 12 items. This score ranged from 0 to 12, with higher scores indicating greater experiences of enacted stigma (Cronbach’s α = 0.65; indicating acceptable level of error associated with measuring the enacted stigma score by using 12 items).

*Health behavior* variables included: Alcohol use, drug use, and sexual behavior (engagement in commercial sex, number of sexual partners, type of sexual intercourse, and condom use during last sex); Participation in group sex and sex under the influence of alcohol and/or drugs during the last 6 months. All variables were dichotomized as “yes” or “no” responses.

*Utilization of HIV prevention and testing services* was assessed by assessing the following: Knowledge of HIV testing sites and access to condoms; Awareness of Pre-Exposure Prophylaxis (PrEP); Condom use during last sex and PrEP use within the last 12 months. All variables were dichotomized as “yes” or “no” responses.

### Data analysis

We conducted descriptive analyses to determine the distribution of key variables among participants. Bivariate analyses were used by using univariate logistic regression to examine associations between independent variables and two primary outcomes: (1) HIV status (HIV positive vs. negative) and (2) HIV testing uptake within the last six months. Multivariable logistic regression was employed to assess the adjusted associations with the two outcomes separately. Variables were selected for inclusion in the models based on their statistical significance in the bivariate analysis and their relevance to the pre-specified hypotheses. This approach allowed for the adjustment of potential confounding factors and the identification of independent predictors of HIV testing experience within the last six months and HIV status. We report adjusted odds ratios (aORs) with 95% confidence intervals (CIs). For the assessment of reliability of the measurement of enacted stigma, we calculated internal consistency reliability coefficient (Chronbach’s α) for the respective 12-items score. Statistical analyses were performed using the Statistical Package for the Social Sciences (SPSS, version 27).

## Results

### Main characteristics of study participants

Table 1 outlines the main characteristics of the study participants.

**Table 1:** Sample characteristics.

	Total sample (N = 95)	Transgender Women (49.5%, n = 47)	Transgender Men (7.3%, n = 7)	Non-Binary (43.2%, n = 41)
**Sociodemographic characteristics** **(total sample N = 95)**				
**Mean Age (SD)**	27.24 (8.52)	29.70 (9.69)	30.43 (8.66)	23.88 (5.56)
**Education**				
** **High school or lower	36.8% (35/95)	44.7% (21/47)	28.6% (2/7)	29.3% (12/41)
** **College, university and higher	63.2%(60/95)	55.3% (26/47)	71.4% (5/7)	70.7% (29/41)
**Emplyment status**				
** **Employed	37.9% (36/95)	25.5% (12/47)	57.1% (4/7)	48.8% (20/41)
** **Self employed	20.0% (19/95)	23.4% (11/47)	28.6% (2/7)	14.6% (6/41)
** **Part-time work	20.0% (19/95)	4.3% (2/47)	0% (0/7)	9.8% (4/41)
** **Paid sex services	37.9% (36/95)	68.1% (32/47)	0% (0/7)	9.8% (4/41)
**Monthly income**				
** **<=500 GEL (180€) **	37.0% (34/92)	28.3% (13/46)	42.9% (3/7)	46.2% (18/39)
** **>500 GEL (180€)	63.0% (58/92)	71.7% (33/46)	57.1% (4/7)	53.8% (21/39)
**Living conditions**				
** **Lives alone	34.7% (33/95)	48.9% (23/47)	28.6% (2/7)	19.5% (8/41)
** **Lives in Tbilisi	76.8% (73/95)	63.8% (30/47)	57.1% (4/7)	95.1% (39/41)
** **Had permanent place of residence over the past 3 months	25.3% (24/95)	25.5% (12/47)	14.3% (1/7)	26.8% (11/41)
**HIV risk behaviors (total sample N = 95)**				
** **Was involved into commercial sex	45.7% (43/94)	78.3% (36/46)	0% (0/7)	17.1% (7/41)
** **Ever participated in a group sex	78.9% (45/57)	89.7% (26/29)	0% (0/1)	70.4% (19/27)
** **Had a sex under the influence of alcohol and/or drugs during the last 6 months	59.1% (55/93)	59.6% (28/47)	14.3% (1/7)	66.7% (26/39)
** **Used a condom during last sex	78.0% (71/91)	87.2% (41/47)	14.3% (1/7)	78.4% (29/37)
** **Used any non-injection drugs during last 12 months	58.9% (56/95)	48.9% (23/47)	42.9% (3/7)	73.2% (30/41)
** **Used injection drug during last 12 months	2.1% (2/95)	2.1% (1/47)	0% (0/7)	2.4% (1/41)
** **Ever used any injecting drugs	5.3% (5/95)	6.4% (3/47)	0% (0/7)	4.9% (2/41)
**Stigma/Discrimination/violence (total sample N = 95)**				
** **During last 12 months because of gender identity experienced:				
** **Psychological pressure	73.7% (70/95)	78.7% (37/47)	85.7% (6/7)	65.9% (27/41)
** **Insult and humiliation	75.8% (72/95)	78.7% (37/47)	57.1% (4/7)	75.6% (31/41)
** **Blackmail (including extortion, outing, threats, intimidation)	17.9% (17/95)	23.4% (11/47)	0% (0/7)	14.6% (6/41)
** **Injury/physical pain	26.3% (25/95)	38.3% (18/47)	0% (0/7)	17.1% (7/41)
** **Physical pursuit	25.3% (24/95)	31.9% (15/47)	0% (0/7)	22.0% (9/41)
** **Failure to get medical care in case of need	5.3% (5/95)	10.6% (5/47)	0% (0/7)	0% (0/41)
** **Was denied a job	20.0% (19/95)	27.7% (13/47)	28.6% (2/7)	9.8% (4/41)
** **Coercion to sex without a condom	13.7% (13/95)	17.0% (8/47)	0% (0/7)	12.2% (5/41)
** **Sexual violence/ an attempted rape	20.0% (19/95)	23.4% (11/47)	0% (0/7)	19.5% (8/41)
** **Rape	6.3% (6/95)	10.6% (5/47)	0% (0/7)	2.4% (1/41)
** **Coercion to sex in a form that is unacceptable (including in perverted forms, with the use of physical violence, group sex)	13.7% (13/95)	14.9% (7/47)	0% (0/7)	14.6% (6/41)
** **Forced use of drugs or alcohol	3.2% (3/95)	4.3% (2/47)	0% (0/7)	2.4% (1/41)
** **Mean Enacted stigma score (SD)	3.01 (2.00)	3.6 (2.15)	1.71 (0.49)	2.56 (1.77)
**The knoweldege/Use of HIV preventive services (total sample N = 95)**				
** **Knows where to get HIV testing	94.7% (90/95)	100% (47/47)	85.7% (6/7)	90.2% (37/41)
** **Ever heard about PrEP	81.1% (77/95)	80.9% (38/47)	85.7% (6/7)	80.5% (33/41)
** **Received condoms for free during the last 12 months	85.3% (81/95)	95.7% (45/47)	71.4% (5/7)	75.6% (31/41)
** **Participated in PreP during last 12 months	28.6% (22/77)	31.6% (12/38)	0% (0/6)	30.3% (10/33)
**HIV testing experience (total sample N = 95)**				
** **Had HIV testing experience during the last 6 months	76.8% (73/95)	89.4% (42/47)	57.1% (4/7)	65.9% (27/41)
** **No HIV testing experience during the last 6 months	23.2% (22/95)	10.6% (5/47)	42.9% (3/7)	34.1% (14/41)
**Prevalence of HIV, HCV, HBV and Syphilis (tested for HIV N = 84, tested for HCV/HBV//Syphilis N = 85)**				
** **HIV	24.1% (20/83)	40.5% (17/42)	0% (0/6)	8.6% (3/35)
** **HBV	1.2% (1//85)	2.3% (1/43)	0% (0/7)	0% (0/35)
** **HCV	9.4% (8/85)	14.0% (6/43)	0% (0/7)	5.7% (2/35)
** **Syphilis	8.2% (7/85)	16.3% (7/43)	0% (0/7)	0% (0/35)

*Denominators varied across variables due to differences in participant responses. Only valid responses were included in the analysis for HIV, HCV, HBV and Syphilis testing results, group sex, monthly income, sex under alcohol/drug and PrEP use.

**Monthly income is dichotomized on the basis of monthly average per capita income in Georgia, which was 521 (188€) GEL in 2023. https://www.geostat.ge/ka/modules/categories/50/shinameurneobebis-shemosavlebi.

Overall, 95 participants were included in this study (S1 Database), with 49.5% identifying as transgender women (n = 47), 7.3% as transgender men (n = 7), and 43.2% as non-binary individuals (n = 41). The mean age of the total sample was 27.24 years (SD = 8.52). Non-binary individuals had a lower mean age (23.88 years, SD = 5.56) compared to both transgender men (30.43 years, SD = 8.68) and transgender women (29.70 years, SD = 9.69).

Overall, 63.2% of participants (N = 60) had a college or higher education, with the highest proportion among transgender men (71.4%, n = 5) and non-binary individuals (70.7%, n = 29), while transgender women had the lowest percentage (55.3%, n = 26). Employment varied across groups: while 37.9% of the total sample (N = 36) were employed, employment was most common among transgender men (57.1%, n = 4) and least common among transgender women (25.5%, n = 12). The income received from paid sex services was also noted by 37.9% (N = 36) of the participants, with transgender women reporting the highest involvement (68.1%, n = 32), while no transgender men and only a small proportion of non-binary individuals (9.8%, n = 4) reported earning income from sex work. Monthly income of 500 GEL (180€) or less was reported by 37.0% of participants (N = 34), with the highest proportion among non-binary individuals (46.2%, n = 18) and transgender men (42.9%, n = 3), while transgender women had the lowest proportion (28.3%, n = 13). Living alone was reported by 34.7% of participants (N = 33), highest among transgender women (48.9%, n = 23) and lowest among non-binary individuals (19.5%, n = 8).

In terms of risky sexual behaviors, engagement in commercial sex work was reported by a significant portion of transgender women (78.3%, n = 36). Group sex participation was common among transgender women (89.7%, n = 26) and non-binary individuals (70.4%, n = 19), while transgender men did not engage in commercial sex work or group sex. Condom use during the last sexual encounter was reported by 78.0% of the sample (N = 71), with the highest proportion among transgender women (87.2%, n = 41) and non-binary individuals (78.4%, n = 29), while transgender men had the lowest reported condom use (14.3%, n = 1). In terms of substance use, non-injection drug use in the past year was reported by 58.9% of the entire sample (N = 56), with the highest proportion among non-binary individuals (73.2%, n = 30) and the lowest among transgender men (42.9%, n = 3). Injection drug use was rare among the entire sample (2,1%, N = 2) and not reported by transgender men. Regarding substance use during sexual activity, 59.1% (N = 55) of the sample reported having sex under the influence of alcohol and/or drugs in the past 6 months, with the highest proportion among non-binary individuals (66.7%, n = 26) and transgender women (59.6%, n = 28), and the lowest among transgender men (14.3%, n = 1).

Participants provided information on their experiences of stigma, discrimination, and violence in the past 12 months due to their gender identity. Psychological pressure, including being gossiped about and subjected to intrigue due to gender identity was reported by 73.7% of participants (N = 70), most frequently among transgender men (85.7%, n = 6) and least among non-binary individuals (65.9%, n = 27). Instances of insult and humiliation were common (75.8%, N = 72), with similar proportions for transgender women (78.7%, n = 37) and non-binary individuals (75.6%, n = 31), but lower among transgender men (57.1%, N = 4). Injury or physical pain was reported by 26.3% of participants (N = 25), with the highest prevalence among transgender women (38.3%, n = 18). Experiences of sexual violence varied across groups: one-fifth of participants (20.0%, N = 19) reported experiencing sexual violence/ an attempted rape, with transgender women again being the most affected group (23.4%, n = 11). A similar pattern was observed for experiences of coercion to engage in sex without a condom. No instances of physical violence, including sexual violence, were reported by transgender men. Coercion to sex in unacceptable forms was reported by 13.7% (N = 13) of participants, with similar proportions among transgender women (14.9%, n = 7) and non-binary individuals (14.6%, n = 6), while no transgender men reported such experiences. Forced drug or alcohol use was reported by 3.2% (N = 3), occurring among 4.3% (n = 2) of transgender women and 2.4% (n = 1) of non-binary individuals, with no cases among transgender men. Denial of medical care was exclusively reported by transgender women (10.6%, n = 5). The mean enacted stigma score was 3.01 (SD = 2.00), with transgender women reporting the highest score (M = 3.6, SD = 2.15), followed by non-binary individuals (M = 2.56, SD = 1.77), and transgender men (M = 1.71, SD = 0.49).

The majority of participants (94.7%, n = 90) were aware of where to access HIV testing, with transgender women having the highest awareness (100%, N = 47) and transgender men the lowest (85.7%, N = 6). Receiving condoms for free in the past year was reported by 85.3% of participants (N = 81), again highest among transgender women (95.7%, N = 45) and lowest among transgender men (71.4%, N = 5). Awareness of PrEP was also high (81.1%, N = 77) and consistent across groups, yet only 28.6% (N = 22) had used PrEP during the same period, with the highest participation among transgender women (31.6%, n = 12) and non-binary individuals (30.3%, n = 10), while no transgender men reported PrEP use. HIV testing experience during the last 6 months varied across subgroups, with 89.4% (n = 42) of transgender women, 57.1% (n = 4) of transgender men, and 65.9% (n = 27) of non-binary individuals reporting recent HIV testing.

Prevalence of HIV, HCV, HBV and syphilis: Of the 95 participants, 88.4% (n = 84) consented to HIV testing, while 85 participants agreed to be tested for HCV, HBV, and syphilis. Among those tested, one individual received an uncertain HIV test result. Eleven participants declined rapid testing for all four infections. Among those who underwent HIV testing, the overall prevalence was 24.1% (N = 20), with the highest prevalence observed among transgender women (40.5%, n = 17) and the lowest among non-binary individuals (8.6%, n = 3). The prevalence of HCV was 9.4% (N = 8), again highest among transgender women (14.0%, n = 6) and lowest among non-binary individuals (5.7%, n = 2). HBV (1.2%, N = 1) and syphilis (8.2%, N = 7) were detected exclusively among transgender women. None of the transgender men tested positive for any of these infections. Co-infections were observed in several participants: five had both HIV and HCV, six had HIV and syphilis, and three tested positives for HIV, HCV, and syphilis. No cases of HBV co-infection were identified.

### Factors associated with HIV positive status

Among the 83 participants screened for HIV during the survey, several factors were associated with HIV positive status in the bivariate analysis, as presented in Table 2.

**Table 2:** Factors associated with HIV status.

Variable	HIV Negatives	HIV Positives	ORa (95% CI)	aORb (95% CI)
**Sociodemographic characteristics**				
**Gender identity**				
Non-binary	91.4% (32/35)	8.6% (3/35)	Ref	
Transgender women	59.5% (25/42)	40.5% (17/42)	**7.25 (1.91-27.54)**	1.71 (0.26-11.52)
Transgender men	100.0% (6/6)	0.0% (0/6)	0.91 (0.83-1.01)	
**Mean Age (SD)**	26.71 (9.03)	30.50 (7.96)	1.04 (0.99-1.1)	1.06 (0.98-1.14)
**Education**				
College, university and higher	82.4% (42/51)	17.6% (9/51)	Ref	
High school or lower	65.6% (21/32)	34.4% (11/32)	2.44 (0.88-6.81)	1.79 (0.5-6.45)
**Employed/self-employed**				
Yes	86.7 (26/30)	13.3% (4/30)	Ref	
No	69.8% (37/53)	30.2% (16/53)	2.81 (0.84-9.38)	
**Monthly income**				
<=500 GEL	87.5% (28/32)	12.5% (4/32)	Ref	
>500 GEL	68.8% (33/48)	31.3% (15/48)	3.18 (0.95-10.70)	3.13 (0.63-15.45)
**Lives currently alone**				
No	80.0% (44/55)	20.0% (11/55)	Ref	
Yes	67.9% (19/28)	32.1% (9/28)	1.90 (0.68-5.32)	
**Permanent place of residence over the past 3 months**				
Yes	85.7% (18/21)	14.3% (3/21)	Ref	
No	72.6% (45/62)	27.4% (17/62)	2.27 (0.59-8.69)	
**Stigma, discrimination and violence experience during last 12 months**				
**Mean Enacted Stigma Score (SD)**	2.87 (1.94)	3.75 (2.17)	1.23 (0.96-1.57)	1.14 (0.84-1.53)
**HIV risk behaviors**				
**Had a sex under the influence of alcohol and/or drugs during the last 6 months**				
No	81.8% (27/33)	18.2% (6/33)	Ref	
Yes	71.4% (35/49)	28.6% (14/49)	1.80 (0.61-5.30)	
**Involved in commercial sex**				
No	89.4% (42/47)	10.6% (5/47)	Ref	
Yes	58.3% (21/36)	41.7% (15/36)	**6.00 (1.92-18.75)**	2.59 (0.49-13.63)
**Used a condom during last sex**				
No	94.4% (17/18)	5.6% (1/18)	Ref	
Yes	69.8% (44/63)	30.2% (19/63)	7.34 (0.91-59.19)	3.47 (0.31-40.55)
**Used any non-injection drugs during last 12 months**				
Yes	77.4% (41/53)	22.6% (12/53)	Ref	
No	73.3% (22/30)	26.7% (8/30)	1.24 (0.44-3.49)	
**Ever used any injection drugs**				
Yes	80.0% (4/5)	20.0% (1/5)	Ref	
No	75.6% (59/78)	24.4% (19/78)	1.29 (0.14-12.24)	4.04 (0.27-59.88)

OR^a^—unadjusted odds ratio.

aOR^b^—adjusted odds ratio controlling for age, gender identity, income, education, involvement in commercial sex, use of a condom during the last sex, enacted stigma score, ever injecting drugs.

Transgender women had significantly higher odds of being HIV-positive compared to non-binary individuals (OR = 7.25, 95% CI: 1.91–27.54). In contrast, none of the transgender men in the study were HIV-positive. Other sociodemographic factors showed higher HIV prevalence mostly among disadvantaged groups: participants with a high school education or lower had a higher prevalence of HIV compared to those with a college or university education. Similarly, those who were unemployed, lived alone, or lacked a permanent place of residence in the past three months had higher HIV prevalence compared to their counterparts. In contrast, participants with a monthly income above 500 GEL had higher HIV prevalence than those earning 500 GEL or less. However, these associations did not reach statistical significance.

Regarding stigma and discrimination experienced in the past 12 months, individuals living with HIV had higher enacted stigma composite scores (Mean = 3.75, SD = 2.17) compared to HIV-negative individuals (Mean = 2.87, SD = 1.94). However, the association between higher stigma scores and HIV-positive status did not reach statistical significance in bivariate analysis (OR=1.23, 95% CI: 0.96–1.57).

Among HIV risk behaviors, one factor was significantly associated with HIV-positive status. Participants involved in commercial sex had six times higher odds of being HIV-positive compared to those who were not engaged in commercial sex (OR = 6.00, 95% CI: 1.92–18.75).

Multivariate Analysis Results: none of the predictor variables reached the level of significance in the multivariate analysis (Table 2).

### Factors associated with HIV testing uptake

Among the 95 participants, 76.8% (n = 73) had undergone HIV testing in the last six months (Table 1). In the bivariate analysis several factors were associated with recent HIV testing uptake (Table 3).

**Table 3:** Factors associated with HIV testing experience during the last 6 months.

Variable	Did not take HIV test during the last 6 months % (n/N)	Took HIV test during the last 6 months % (n/N)	OR^a ^(95% CI)	aOR^b ^(95% CI)
**Sociodemographic characteristics**				
**Gender identity**				
Non-binary	34.1% (14/41)	65.9% (27/41)	Ref	
Transgender women	10.6% (5/47)	89.4% (42/47)	**4.36 (1.41-13.48)**	1.46 (0.26-8.21)
Transgender men	42.9% (3/7)	57.1% (4/7)	0.69 (0.13-3.53)	1.3 (0.19-8.63)
**Mean Age (SD)**	27.82 (11.32)	27.07 (7.57)	1.01 (0.95-1.06)	0.97 (0.9-8.62)
**Education**				
College, university and higher	23.3% (14/60)	76.7% (46/60)	Ref	
High school or lower	22.9% (8/35)	77.1% (27/35)	1.03 (0.38-2.77)	0.88 (0.22-3.56)
**Employed/self-employed**				
No	25.4% (15/59)	74.6% (44/59)	Ref	
Yes	19.4% (7/36)	80.6% (29/36)	1.41 (0.51-3.88)	
**Monthly income**				
<=500 GEL	44.1% (15/34)	55.9% (19/34)	Ref	
>500 GEL	12.1% (7/58)	87.9% (51/58)	**5.75 (2.03-16.28)**	3.29 (0.85-12.7)
**Lives currently alone**				
No	30.6% (19/62)	69.4% (43/62)	Ref	
Yes	9.1% (3/33)	90.9% (30/33)	**4.42 (1.20-16.28)**	**5.9 (1.06-32.69)**
**Permanent place of residence over the past 3 months**				
Yes	37.5% (9/24)	62.5% (15/24)	Ref	
No	18.3% (13/71)	81.7% (58/71)	2.68 (0.96-7.44)	
**Stigma, discrimination and violence experience during last 12 months**				
**Mean Enacted Stigma Score (SD)**	1.91 (1.34)	3.34 (2.06)	**1.63 (1.16-2.28)**	**1.76 (1.04-2.97)**
**HIV risk behaviors**				
**Had sex under the influence of alcohol and/or drugs during the last 6 months**				
No	23.7% (9/38)	76.3% (29/38)	Ref	
Yes	20.0% (11/55)	80.0% (44/55)	1.24 (0.46-3.37)	
**Involved in commercial sex**				
No	37.3% (19/51)	62.7% (32/51)	Ref	
Yes	7.0% (3/43)	93.0% (40/43)	**7.92 (2.15-29.15)**	3.32 (0.51-21.38)
**Used a condom during last sex**				
No	35.0% (7/20)	65.0% (13/20)	Ref	
Yes	16.9% (12/71)	83.1% (59/71)	2.56 (0.87-8.04)	
**Used any non-injection drugs during last 12 months**				
Yes	25.0% (14/56)	75.0% (42/56)	Ref	
No	20.5% (8/39)	79.5% (31/39)	1.29 (0.42-3.46)	
**The knowledge/Use of HIV preventive services**				
**Knows where to get HIV testing**				
No	80.0% (4/5)	20.0% (1/5)	Ref	
Yes	20.0% (18/90)	80.0% (72/90)	**16.00 (1.68-152.01)**	9.71 (0.62-153.1)
**Participated in PreP during last 12 months**				
No	22.2% (12/54)	77.8% (42/54)	Ref	
Yes	9.1% (2/22)	90.9% (20/22)	2.86 (0.58-13.99)	

OR^a^—unadjusted odds ratio.

aOR^b^ adjusted odds ratio controlling for age, gender identity, education, income, involved in commercial sex, living alone, enacted stigma score and knowledge about HIV testing facilities.

In the bivariate model individuals with certain sociodemographic characteristics were significantly more likely to have undergone HIV testing in the past six months. Transgender women had over four times the odds of HIV testing compared to non-binary individuals (OR = 4.36, 95% CI: 1.41-13.48). Additionally, those earning more than 500 GEL per month were nearly six times more likely to have tested compared to those with lower incomes (OR = 5.75, 95% CI: 2.03-16.28). Living alone was also associated with increased HIV testing uptake, with individuals who lived alone being more than four times as likely to have tested compared to those living with others (OR = 4.42, 95% CI: 1.20-16.28). Higher enacted stigma score was significantly associated with recent HIV testing (OR=1.63, 95% CI: 1.16–2.28). Among the HIV risky behavior, individuals who involved in commercial sex had nearly eight times the odds of HIV testing compared to those who were not engaged in sex work (OR = 7.92, 95% CI: 2.15-29.15). Knowledge and access to HIV prevention services also influenced testing behavior. Participants who knew where to get an HIV test were significantly more likely to have been tested in the past six months (OR = 16.00, 95% CI: 1.68-152.01).

In the adjusted model, two factors remained significantly associated with recent HIV testing. Participants who lived alone were more likely to have taken an HIV test in the last six months (aOR=5.9, 95% CI: 1.06–32.69) and a higher enacted stigma score was also positively associated with recent HIV testing (aOR=1.76, 95% CI: 1.04–2.97).

## Discussion

Our study sheds new light on the burden of HIV (24.1%) in an understudied group of transgender people in Georgia, a country within the EECA region with a growing HIV epidemic. While data on HIV prevalence among transgender people in the EECA region remain limited, a similar prevalence rate was reported in Kazakhstan, highlighting the widespread vulnerability of this group [14]. An assessment of HIV strategic information in seven countries in Eastern Europe and Central Asian (EECA) regions (Armenia, Belarus, Georgia, Kyrgyzstan, North Macedonia, Tajikistan and Estonia) revealed that the largest gap is related to lack of information and research on transgender populations [15].

Our study highlights that transgender women represent the subgroup most affected by HIV (40.5%) within the broader transgender community. This finding aligns with global context that consistently shows transgender women bear a disproportionate burden of HIV compared to other transgender identities, such as transgender men or nonbinary individuals [6]. Studies from the United States [16] and Brazil [17] have also reported high HIV prevalence rates among transgender women, at 29% and 32.1%, respectively.

Despite the high HIV burden in this population, gaps remain in the utilization of HIV testing and prevention services. While transgender women reported the highest HIV testing rates (89.4% in the last six months), transgender men had the lowest (57.1%), highlighting disparities in service uptake. Similar patterns of high HIV testing uptake have been observed in studies from the United States (78.6% HIV testing uptake among transgender individuals [18] and Thailand (75% testing uptake among transgender women [19]. These findings underscore the need for targeted interventions to improve HIV testing access, particularly among subgroups with lower utilization rates. Regarding PrEP uptake, although Georgia has provided free PrEP to high-risk groups since 2017, our study found that only 28.6% of participants had accessed PrEP in the last 12 months. This suggests that despite its availability, barriers to PrEP access persist within the transgender community. Further efforts are needed to improve awareness and utilization of PrEP through tailored outreach and community-based interventions.

Our study identified two significant predictors of HIV testing uptake in the multivariate analysis: enacted stigma and living alone. Enacted stigma, measured through various experiences of discrimination, emerged as a key factor influencing HIV testing. Transgender individuals who reported higher enacted stigma scores were more likely to have undergone HIV testing. Breaking down individual enacted stigma indicators, our study found that 73.7% of participants reported experiencing psychological pressure, while 75.8% experienced insult and humiliation due to their gender identity. Transgender women were the most affected by physical violence (38.3%) and sexual violence (23.4%), reinforcing their heightened vulnerability. While stigma is often considered a barrier to healthcare access [4] our findings suggest that experiences of discrimination and violence may, in some cases, act as a catalyst for HIV testing, possibly due to heightened risk perception [20] or engagement with supportive community organizations.

Another important predictor of HIV testing according to our study results was living alone. Our analysis showed that individuals who lived alone were more likely to undergo HIV testing. This may be due to experiences of discrimination and rejection by family members and friends, prompting some to choose living alone for personal safety and to avoid further marginalization [21]. This finding aligns with the result that experiences of discrimination and violence were positively associated with HIV testing. This form of social exclusion may drive transgender individuals to create independent, safer living situations in which they can better manage their health and well-being. Further research is needed to explore these associations in greater depth, particularly to understand the underlying mechanisms through which social exclusion and independent living influence HIV testing behaviors among transgender individuals.

Our study has several limitations. The higher HIV prevalence observed among transgender individuals, particularly transgender women, is likely influenced by selection bias inherent to our sampling approach [22]. This study utilized snowball sampling, meaning that participation depended on peer referrals and outreach efforts rather than random selection. As a result, certain subgroups of transgender individuals may have been overrepresented. For instance, transgender women engaged in sex work—who are more connected to HIV prevention services and peer networks—were more likely to be recruited, potentially contributing to the higher observed HIV prevalence. Conversely, more socially isolated transgender individuals, including those not engaged in sex work or prevention programs, may have been underrepresented, which could impact the generalizability of our findings [23]. In addition, the way gender identity was captured and categorized in the study—into transgender women, transgender men, and non-binary individuals—may not fully reflect the complexity of participants’ identities. Specifically, we were unable to disaggregate data on transmasculine individuals within the non-binary group. This limits our ability to explore and present the health-related experiences of transmasculine people, who may share similar vulnerabilities and healthcare needs with transgender men.

Additionally, self-reported data on sexual behaviors and experiences of discrimination are subject to social desirability bias, potentially underestimating the true prevalence of these factors [24]. A significant limitation of this study is the small sample size, which may impact the reliability and generalizability of the findings. The small sample size restricts the ability to conduct more nuanced subgroup analyses, which could provide deeper insights into specific factors influencing HIV risk among different subgroups within the transgender community. In addition, our sample was drawn from three cities in Georgia (Tbilisi, Batumi, and Kutaisi), and while these cities represent different regions, the transgender population in rural areas or other parts of the country may have different HIV risk profiles.

Despite these limitations, snowball sampling remains a widely used and pragmatic approach for research involving hard-to-reach and marginalized populations, particularly in settings where stigma and discrimination hinder recruitment through conventional methods [22,25]. It has been successfully applied in transgender health research, particularly in HIV-related studies, where traditional sampling strategies may not be feasible [26].

## Conclusions

Our study reveals a high burden of HIV among transgender individuals in Georgia, particularly transgender women, combined with significant gaps in the utilization of HIV prevention services. While HIV testing uptake was relatively high among some subgroups, barriers remain, particularly for transgender men. The association between enacted stigma and HIV testing uptake underscores the complex relationship between discrimination and healthcare-seeking behaviors. Similarly, the finding that individuals who live alone were more likely to undergo HIV testing suggests that social isolation may drive proactive engagement with healthcare services. Further research is needed to explore the intersection of stigma with other barriers to HIV testing and care, particularly structural and socioeconomic factors. By implementing targeted, evidence-based interventions, public health efforts can work toward reducing the burden of HIV and improving health outcomes for transgender individuals in Georgia and the broader EECA region.

## Supporting information

S1 Database Raw, anonymized qualitative data excerpts used in the analysis of barriers and facilitators to HIV testing among transgender people in Georgia. (XLSX)
